# Personalized Test Bolus MSCT Protocol for Optimal Coronary Sinus Venous Visualization in Candidates for Cardiac Resynchronization Therapy

**DOI:** 10.3390/jcm15135022

**Published:** 2026-06-27

**Authors:** Stepan Zubarev, Sergey Rud’, Mikhail Chmelevsky, Vera Stepanova, Aleksandr Sinitca, Lev Malishevskii, Tatiana Chumarnaya, Olga Solovyova, Dmitry Lebedev

**Affiliations:** 1 Department of Cardiac Electrophysiology, Almazov National Medical Research Center, Saint-Petersburg 197341, Russia; lev.m.ecg@gmail.com (L.M.); lebedevdmitry@mail.ru (D.L.); 2 Institute of Immunology and Physiology, Ural Branch of the Russian Academy of Sciences, Yekaterinburg 620078, Russia; chumarnaya@gmail.com (T.C.); o.solovyova@iip.uran.ru (O.S.); 3 Department of Function Diagnostics, Saint Petersburg State University, Saint-Petersburg 199034, Russia; boxmch@gmail.com; 4 Department of Radiology, Almazov National Medical Research Center, Saint-Petersburg 197341, Russia; rsd@mail.ru; 5 Department of Function Diagnostics, Almazov National Medical Research Center, Saint-Petersburg 197341, Russia; 6 Department of Function Diagnostics, Pavlov First Saint Petersburg State Medical University, Saint-Petersburg 197022, Russia; 7 Department of Cardiac Electrophysiology, I.I. Mechnikov North-Western State Medical University, Saint-Petersburg 191015, Russia; stepanova.vera.vl@gmail.com; 8 Department of Radio Engineering Systems, Saint-Petersburg Electrotechnical University “LETI”, Saint-Petersburg 197022, Russia; amsinitca@etu.ru; 9 Institute of Natural Sciences and Mathematics, Ural Federal University, Yekaterinburg 620002, Russia

**Keywords:** multislice computer tomography, test bolus, coronary sinus visualization, computer tomography protocol, medical imaging, cardiac resynchronization therapy

## Abstract

**Background/Objectives**: A thorough understanding of the anatomy of the coronary sinus (CS) and its tributaries provides valuable information for selecting the optimal left ventricular lead and may even prompt reconsideration of the endovascular implantation strategy when planning cardiac resynchronization therapy (CRT). Currently, there is no personalized multislice computer tomography (MSCT) protocol for CS veins visualization that is suitable for all diverse candidates. **Methods**: a single-center prospective study included 74 various adult patients with recommendation class I and IIA for CRT. Prior to implantation, all participants underwent contrast MSCT to evaluate the CS veins. The first aspect of MSCT involved the administration of a test bolus to enable the automated calculation of the time-to-peak contrast opacification within the ascending aorta. The second aspect consisted of adding a fixed extra value of 20 s. The resulting sum was then used as the final delay to scan CS veins. The final cardiac acquisition was performed with prospective gating and manual phase in the range of 200–400 ms. The contrast media involved a standard iodine concentration of 300 mg I/mL, an injection rate not exceeding 4.5 mL/s, and a total contrast dose of up to 115 mL. **Results**: in all patients presented, all first-order CS branches were detected. The analysis found no statistically significant effect of heart rate and heart rhythm on the quality of venous visualization. The coefficient of determination (r_s_^2^) revealed that only 28.9% of the rank variability in time-to-peak contrast opacification can be explained by Hounsfield unit. This underscores that only the test-bolus protocol with definitively calculated time delay can ensure a personalized optimal enhancement of CS veins. **Conclusions**: a personalized, detailed test bolus MSCT protocol for coronary sinus veins visualization is presented. Multi-vendor, multi-center studies are warranted to confirm the generalizability and external validity of the proposed MSCT protocol.

## 1. Introduction

Despite the active trend toward conduction system pacing, it is important to understand that this approach is not a panacea for all cases. This is especially apparent in patients with atypical left bundle branch block, intraventricular conduction disturbances, or markedly dilated hearts [1,2,3]. Under these circumstances, resynchronization with a single electrode—even when conduction system capture is achieved—proves challenging. This can be attributed to several factors. Firstly, the geometry of the ventricle’s changes, becoming more spherical, which disrupts the normal sequence of contraction. Simultaneously, the distance between cardiomyocytes increases. Secondly, fibrous degeneration of the myocardium occurs, impairing normal impulse conduction. Consequently, isolated conduction system pacing will not be able to completely displace or negate the role of an implanted left ventricle (LV) lead placed via the coronary sinus (CS). The use of conventional cardiac resynchronization therapy (CRT) technique or the growing left optimized therapy (LOT-CRT) with an LV lead will undoubtedly continue. In this light, knowing the anatomy of the LV venous tree, represented by the CS and its tributaries, is still a crucial point for CRT.

The current benchmark for veins detection is occlusive balloon contrast venography, which is performed directly in the operating room during CRT implantation. In this regard, information on the anatomy is only available intraoperatively. It is well known that the anatomy of the CS veins exhibits significant interpatient variability [4,5,6]. In the presence of certain anatomical features, it only becomes apparent during the implantation procedure that a different surgical approach would have been preferable (e.g., due to a tortuous ostial CS segment or other challenges) [7,8,9,10]. It is highly desirable to know about CS vein anatomy already at the planning stage in the preoperative period. Hence, it would allow the choice of surgical approaches, the set of tools to avoid fruitless implantation of LV. In this regard, the question of CS veins anatomy can be answered preoperatively by multislice computer tomography (MSCT) as a stand-alone study [11,12] or as part of electrocardiographic imaging (ECGI) [13]. ECGI appears to be a rational three-dimensional imaging technology. It combines tomography and electrocardiography (ECG). The ECG component addresses the question of the location of the electrical late zone, while the tomographic component identifies the target vein for that zone.

Various scanning protocols with different parameters for the assessment of the CS veins have been documented in the literature. There are evident advantages and disadvantages associated with each of the proposed approaches. There is no protocol that remains universally applicable to all patients.

Firstly, there are the bolus tracking and test bolus techniques. The bolus tracking approach is based on the automatic initiation of scanning once the contrast agent reaches a predefined attenuation threshold in Hounsfield unit (HU) within the selected region of interest [14,15,16,17,18]. The test bolus technique enables a personalized and precise scan timing by characterizing individual circulation times, thereby minimizing variability in CS venous opacification [19]. It would be of interest to explore whether, in most patients, the HU value and the time-to-peak contrast opacification during the test bolus vary independently. Additionally, it would be useful to ascertain whether the time-to-peak is unaffected by patient-specific clinical parameters. Observations of this nature would provide further evidence supporting the use of a test bolus.

Second component: the structure used for test bolus monitoring. There are mentions that the measurement level is placed either in the CS or in the ascending aorta [19]. Using the ascending aorta as a reference seems to be more preferable. When the CS is used as reference, the test bolus time–attenuation curve may contain substantial noise, making it difficult to identify the true time-to-peak contrast opacification for further calculation.

The third component involves the use of retrospective [8,11] or prospective gating [13,15,19]. Both diastolic and systolic acquisition phases are described [14,15,16,18,19]. Prospective gating appears to be more advantageous due to its lower radiation dose.

The fourth aspect is the heart rhythm and rate. Several studies are performed only with sinus rhythm and at a heart rate of around 65 beats per minute [16,20,21]. Obviously, the protocol should allow scanning any patient. And the final quality of CS veins visualization should not be dependent on heart rate and rhythm during contrast enhancement.

The fifth component is the contrast medium and its dosage. Certainly, a high iodine concentration of agent will yield superior image quality. However, it should be considered to use a standard iodine concentration of 300 milligrams of iodine per milliliter (300 mg I/mL) to ensure better patient tolerance. In the literature, the dose ranges from 100 to 120 milliliters (mL), which is generally well-tolerated by patients without adverse effects [11,13].

Last but not least is the contrast injection rate. In a several studies, the rate is 5–6 mL per second (mL/s) [19,20]. It should be understood that not every patient’s antecubital vein will be suitable in diameter for catheter placement to withstand such a flow rate. Therefore, the rate should not exceed 4.5 mL/s to allow placement of a smaller diameter catheter.

Overall, the literature review indicates that there remains scope for improving current approaches to CS opacification during MSCT.

## 2. Materials and Methods

The objective of this study was to develop and evaluate a standardized MSCT scanning protocol for reliable visualization of the CS veins across candidates for CRT implantation with different heart rates and rhythm patterns.

### 2.1. Study Population

This single-center prospective study enrolled 74 adult patients with chronic heart failure despite guideline-directed medical therapy who met the criteria for CRT implantation. The study was conducted from December 2022 to December 2025. As this work was designed primarily for protocol development, the cohort of 74 patients was intended to provide preliminary, hypothesis-generating data rather than to confirm a predefined effect. Also, the sample size was determined by the amount of funding allocated for this study. Inclusion criteria had several items. Eligible adult candidates over 18 years of age encompassing both Class I and Class IIa CRT indications [22] were included in the investigation. All participants signed informed consent. Patients did not have standard contraindications to contrast-enhanced MSCT. Only patients with kidney function stages 1 to 3A inclusive, according to the estimated glomerular filtration rate-based classification, were eligible for the investigation [23]. A methodological limitation for protocol was patient body weight exceeding 200 kg, or a chest circumference greater than the width of the MSCT gantry aperture, which makes scanning impossible. Another relative limitation was the presence of extensive orthopedic metal implants in the thoracic spine region, which may generate additional artifacts and adversely affect image quality. Generative artificial intelligence was not used in this paper for dataset generation or augmentation.

### 2.2. Multislice Computer Tomography

**Figure 1:**
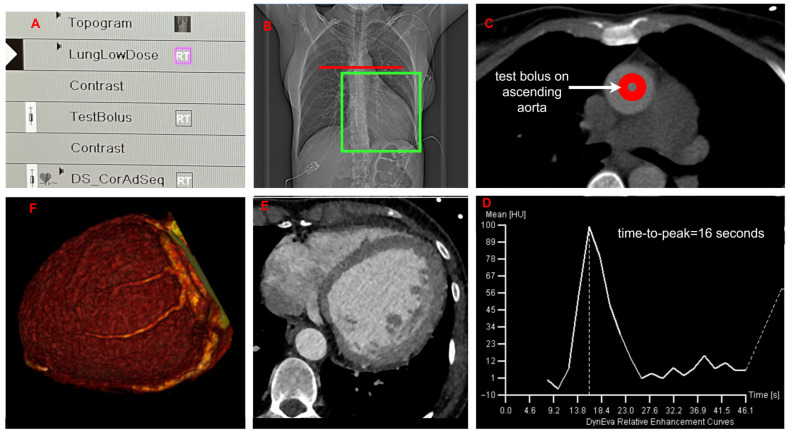
Tomography pipeline. (**A**)—all sequential steps; (**B**)—Topogram; red line—carina level for test bolus; green box—heart borders; (**C**)—adding a marker in the ascending aorta in DynEva Siemens application to form a graphic; (**D**)—automatic detection time-to-peak in the ascending aorta on the curve during the contrast test bolus; (**E**)—DS_CorAdSeq (venous phase during contrast enhancement to visualize coronary sinus with tributaries); (**F**)—3D image of the heart with contrast-enhanced coronary sinus veins.

Prior to CRT implantation, all participants underwent contrast-enhanced MSCT (SOMATOM Force, Siemens Healthineers, Germany). It included the following steps: Topogram; LungLowDose (optional series); test bolus; DS_CorAdSeq (Figure 1).

#### 2.2.1. Topogram

The Topogram was performed on the patient’s breath. The hands position was up. Set the start of the scan from the upper thoracic aperture. Parameters for the Topogram were configured according to Table 1.

#### 2.2.2. LungLowDose Thorax Series

The field of view was selected so that the skin surface of the thorax area was completely displayed during image reconstruction. The scanning area should include the entire chest area from the thorax aperture to the level of the 12th rib. Parameters for performing a non-contrast LungLowDose series are described in Table 2.

Performing this series is optional. If, ultimately, only an evaluation of the CS veins is planned, this series can be omitted, and the radiologic technologist can proceed directly to the test bolus. However, if the study is supplemented with ECGI, this series is required to evaluate torso geometry. Which is an important component required to solve the mathematical problem involved in constructing ventricular activation maps [24]. Ultimately, this makes it possible to detect the target electrical zone of late activation [13,25,26]. In the current study, we performed this series for all patients to receive geometry of a torso for further identification the zone of late activation via ECGI.

#### 2.2.3. Test Bolus

Before this step was performed, it was ensured that the prepared intravenous contrast delivery system was ready and connected to the injector (Stellant D Dual Syringe, Medrad, Pittsburgh, PA, USA). The test bolus tracking area settled at the carina level (Figure 1B) and configured according to the parameters in Table 3.

We selected the contrast mode ‘injector coupled (start button)’. Since time-to-peak enhancement was assessed in the ascending aorta, it was not advisable to begin scanning patients immediately. In all cases, an 8 s delay was applied between the start of power-injector administration and the initiation of test-bolus scanning (Table 3). This time interval was necessary to allow the contrast medium to reach the ascending aorta. We always applied the total number of scans to 23 to ensure that the peak value of contrast opacification in the aorta was not missed (Table 3). In this study, we did not interrupt the acquisition of the complete 23-scan series to subsequently verify the correctness of the aortic contrast-enhancement curve. In general, if the radiologic technologist observes on real-time dynamic images that peak contrast opacification has already been achieved, this may prompt early termination of the series—without completing all 23 repeat scans—to reduce radiation exposure. The parameters for injecting contrasting agent on the auto-dispenser are described in Table 4.

Any contrast medium available in the clinic for routine practice could be employed. We used the contrast agent Iopromid (Ultravist) with a standard iodine concentration of 300 mg I/mL. Naturally, if a contrast medium with an even higher iodine concentration was used, it was possible to obtain even better final images. Contrast was administered through a 20-gauge pink peripheral intravenous catheter in the antecubital vein.

After completing the test bolus, the radiologic technologist had to return to the scanner’s main menu. Then, the recorded test bolus series for this patient was selected and the technologist went to the DynEva application. In this application, we added a marker in the ascending aorta (Figure 1C) and set the 8 s delay from the start of the injection. After this, a graph of the filling curve appeared (Figure 1D). This graph displayed the time-to-peak contrast opacification in the ascending aorta. This value was used in the next step. A screenshot of the curve was saved in the patient’s study report.

#### 2.2.4. DS-CorAdSeq Series (Venous Phase During Cardiac Contrast Enhancement) with ECG Synchronization

The key point in this heart contrast series was the final scanning delay. It equaled the time-to-peak contrast opacification in the ascending aorta (which was calculated during the test bolus step) plus 20 s. This pause was necessary to allow the contrast to collect in the venous system [19]. As it turned out, adding exactly 20 s consistently yielded optimal contrast enhancement of the CS veins.

**Figure 2:**
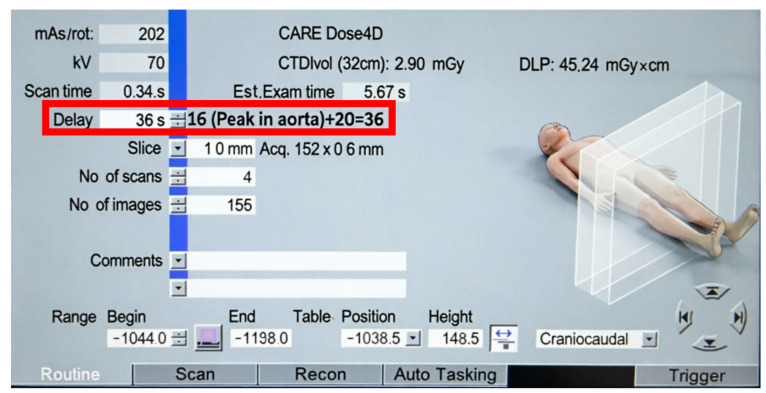
Example to set a final individual calculated scanning delay for DS-CorAdSeq series: 16 (time-to-peak in the aorta) plus 20 (constant extra value) equals 36 s (final delay in this example).

For example, if the time-to-peak contrast opacification in the ascending aorta was 16 s during test bolus, then the final delay for cardiac contrast enhancement in the venous phase was set to 36 s (Figure 2).

DS-CorAdSeq series was done with ECG synchronization and prospective gating to minimize radiation dose. Essential parameters were set according to Table 5.

The parameters for injecting contrasting agent on the auto-dispenser are described in Table 6. The scan protocol with final delay and the auto-injector were started simultaneously. For this purpose, we selected the contrast mode ‘injector coupled (start button)’.

The final view of this series is presented in Figure 1E,F. At the end of the MSCT examination, a number of parameters from the protocol were recorded. The first parameter is Dose Length Product (DLP), which reflected the total radiation exposure received by the patient throughout the entire scanning period. DLP was measured in milligray-centimeters (mGy×cm). Next, Computed Tomography Dose Index Volume (CTDIvol), which characterized the average radiation dose in a standardized phantom (patient model) during a single slice (scan). Then finally, CTDIvol was measured in milligray (mGy). The calibration of the MSCT scanner used in the current study for the calculation of the CTDIvol was based on the size where large = 32 cm and small= 16 cm. Additionally, time-to-peak contrast opacification in the ascending aorta and the corresponding HU value for it were recorded during the test bolus. HU is a conditional unit of the density scale in computed tomography that shows how strongly a tissue attenuates X-ray radiation compared to water. Time-to-peak contrast opacification in the ascending aorta and the corresponding HU was obtained from the automatic curve of the graph in the DynEva application (Figure 1D). Last but not least, it was recorded at what rhythm (sinus, atrial fibrillation, ventricular pacing) and at what heart rate frequency the MSCT was performed.

After performing MSCT the visualization quality of veins was assessed using the following scoring system: −1 point—the vein is absent in the patient; 0 point—the tributary is not visualized; 1 point—the tributary is partially visualized (not throughout its entire length); 2 points—the tributary is fully visualized (throughout its entire length). The presence or absence of a specific vein was confirmed by the results of invasive intraoperative venography during CRT implantation.

### 2.3. Electrocardiography

A 12-lead ECG was carried out to assess the indications for CRT. The QRS complex activation pattern was evaluated based on the Strauss’s criteria [27]. The QRS duration was calculated automatically.

### 2.4. Echocardiography

Each patient was evaluated using echocardiography. The examination included measurement of the left ventricular end-diastolic volume (LV EDV) and left ventricular end-systolic volume (LV ESV) to calculate the left ventricular ejection fraction (LV EF).

### 2.5. Statistical Analysis

Statistical analysis was performed using Statistica version 12 (StatSoft, Tulsa, OK, USA) and R environment (version 4.5.1). The readxl package was used for data import [28], ggplot2 for data visualization [29], the lmtest [30], car [31], MASS [32] for model diagnostics, dplyr [33] and tidyr [34] for data manipulation, rstatix [35] for statistical tests, writexl [36] for data export. To assess the normality of the distribution for continuous variables, the Shapiro–Wilk test was applied. The majority variables exhibited a non-normal distribution (*p* < 0.05); consequently, quantitative data are expressed as the median (minimum–maximum) and the first [Q1] and third [Q3] quartiles. Spearman’s rank correlation coefficient (r_s_) was calculated to determine the strength and direction of the associations. Interpretation of correlation coefficients were established as the following: 0–0.3 indicates a weak correlation; 0.3–0.7 indicates a moderate correlation; and >0.7 indicates a strong correlation.

Venous visualization quality was assessed using an ordinal scale: −1 point—vein anatomically absent in the patient; 0 points—tributary not visualized; 1 point—tributary partially visualized (not throughout its entire length); 2 points—tributary fully visualized (throughout its entire length). The following veins were analyzed: CS main trunk, middle vein, posterior vein, posterolateral vein, lateral vein, anterolateral vein, and anterior vein.

Cardiac rhythm type was considered as a categorical variable with three levels: 0—sinus rhythm; 1—atrial fibrillation (AF); 2—right ventricular pacing (RV pace).

Heart rate was analyzed as a continuous variable (beats per minute).

*Analysis of rhythm type effect*

A two-stage approach was used to evaluate the effect of cardiac rhythm on venous visualization quality. First, logistic regression was performed to assess the influence of rhythm on the probability of anatomical vein absence (score −1). Second, after excluding patients with anatomically absent veins, visualization quality (scores 0, 1, 2) was analyzed using the Kruskal–Wallis test to detect differences among the three rhythm groups. When significant differences were found, post hoc analysis was conducted using Dunn’s test with Bonferroni correction for multiple comparisons.

*Analysis of Heart Rate Effect*

Spearman’s rank correlation coefficient was used to assess the association between heart rate and venous visualization quality. Correction for multiple comparisons was performed using the Bonferroni method.

*Multivariable Analysis*

To evaluate the independent effects of rhythm type and heart rate on venous visualization quality, proportional odds logistic regression was applied, including both predictors in the model.

*Generalized Linear Models for Time-to-Peak in the Ascending Aorta*

Generalized linear models (GLMs) were used to identify factors associated with the time-to-peak in the ascending aorta. The dependent variable, aortic time-to-peak, was treated as a continuous variable and expressed in seconds.

The following variables were included in the model as continuous predictors: age (years), Hounsfield unit (HU) value in the ascending aorta, body mass index (BMI; kg/m^2^), heart rate (bpm), QRS complex duration (ms), and left ventricular ejection fraction (LV EF; %). Categorical predictors were entered as factors, with the following reference categories: gender (male), cardiac rhythm (sinus rhythm), etiology (dilated cardiomyopathy), and New York Heart Association (NYHA) functional class (class II).

Prior to model fitting, multicollinearity among the predictors was assessed using the variance inflation factor (VIF). All VIF values were below the pre-specified threshold of 5, indicating the absence of significant multicollinearity [37].

*Model Selection and Specification*

To determine the appropriate distribution family for GLM, the distribution of the dependent variable was examined in several steps. First, the Shapiro–Wilk test and visual inspection of the distribution were used to assess normality. Given that the data did not follow a normal distribution, four theoretical distributions—normal, gamma, log-normal and Weibull—were compared using the maximum likelihood estimation (MLE) method.

Based on this comparison, the log-normal model (i.e., linear regression on log-transformed data) was selected for subsequent analysis. The model was fitted using the lm() function in R with logarithmically transformed the dependent variable. This transformation ensured the positivity of predicted values and allowed for a convenient interpretation of the regression coefficients in terms of multiplicative effects. Specifically, exp(β) represented the multiplicative factor by which the expected value of the dependent variable changed for a one-unit increase in the predictor, corresponding to a percentage change of (exp(β) − 1) × 100%.

*Model Performance and Diagnostics*

The following metrics were used to assess the quality and validity of the model. The coefficient of determination (R^2^) and adjusted R^2^ were used to quantify the proportion of variance explained by the model on the logarithmic scale. An F-test was performed to compare the fitted model with the null model. Spearman’s rank correlation coefficient (r_s_) was calculated between the observed and predicted values. Additionally, the mean absolute error (MAE) and root mean square error (RMSE) were calculated on the original scale, along with the coefficient of variation of the RMSE (CV-RMSE), expressed as a relative error (%). The Breusch–Pagan test was used to assess homoscedasticity of the residuals, and the Durbin–Watson test was applied to detect the presence of autocorrelation.

*Interpretation of Regression Coefficients*

Due to the logarithmic transformation of the dependent variable, the regression coefficients were interpreted on a multiplicative scale as follows. Positive coefficient (β > 0): a one-unit increase in the predictor is associated with an increase in the expected value of the dependent variable by (exp(β) − 1) × 100%. Negative coefficient (β < 0): A one-unit increase in the predictor is associated with a decrease in the expected value of the dependent variable by (1 − exp(β)) × 100%.

*Statistical Significance*

A conservative significance level of α = 0.01 was adopted instead of the traditional α = 0.05 to minimize the risk of Type I errors (false positives). This threshold was chosen for the following reasons: (1) the clinical consequences of a false association between rhythm type and venous anatomy could lead to unwarranted patient selection for interventional procedures; (2) given the exploratory nature of the study, prioritizing specificity (avoidance of false positives) over sensitivity was deemed more appropriate. At α = 0.01, the probability of erroneously rejecting the null hypothesis is 1%, ensuring a higher level of confidence in the identified associations. Predictors with a *p*-value < 0.01 were considered statistically significant.

## 3. Results

### 3.1. Patients’ Characteristics

Clinical, electrocardiographic and echocardiographic parameters are presented in Table 7.

Values obtained during MSCT are summarized in Table 8. No adverse events were observed in any of the patients following the administration of the contrast agent according to the study protocol.

In all patients, the peak contrast enhancement of the ascending aorta, as assessed by the test-bolus curve in the DynEva application, was distinct. There were no difficulties in interpreting the automated curve or visualizing the maximum aortic peak. This served as a guarantee of success for calculating the final delay in every case.

### 3.2. Veins’ Evaluation

Among the 74 candidates presented, optimal enhancement was achieved during the contrast venous phase with final delay across various rhythms (sinus rhythm, atrial fibrillation, ventricular pacing) and different heart rates. Enhancement was considered optimal when the main CS trunk and at least all the first-order branches were visualized.

#### 3.2.1. Qualitative Assessment of Veins

Table 9 presents the results of the qualitative assessment of veins. Most of the available veins had a grade of 2 points. There was not a single vein that was missed on MSCT but was present on invasive venography.

**Figure 3:**
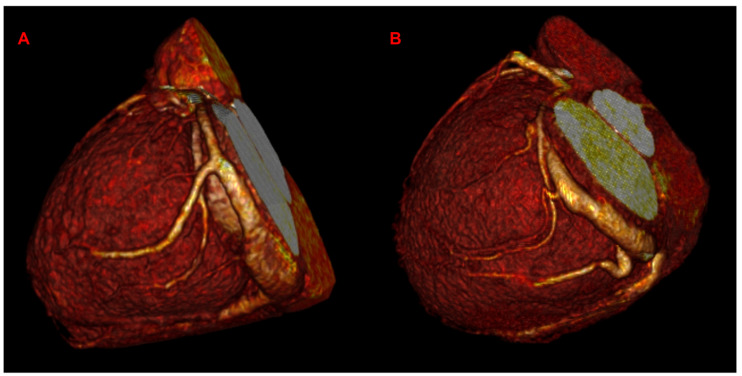
Visualization of veins during different rhythms. (**A**)—patient with atrial fibrillation and heart frequency 112 per minute during acquisition with final scan delay 50 s; (**B**)—patient with sinus rhythm and heart frequency 105 per minute during acquisition with final scan delay 58 s.

Figure 3 demonstrates that CS veins are clearly visible during AF and sinus rhythm with different frequency.

**Figure 4:**
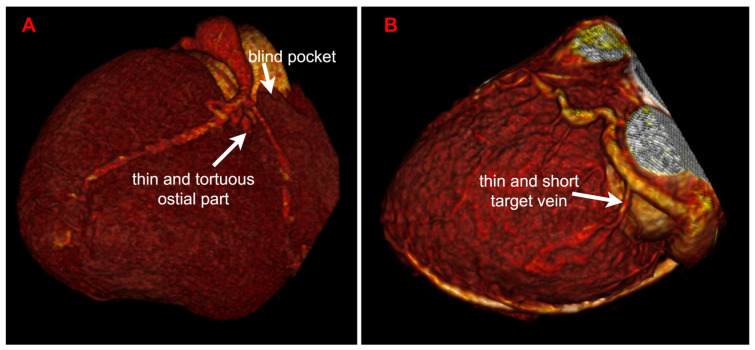
Visualization of cases selected for alternative implantation. (**A**)—blind pocket instead of typical ostial part of coronary sinus. Thin and tortuous ostial part which is connected atypically with right atrium. (**B**)—thin and short target vein (inappropriate for implantation).

The most practical result was that in 8 (10.8%) of 74 patients, the CS veins anatomy was determined to be unsuitable for endovascular LV lead implantation. These patients ultimately received alternative pacing. Figure 4 demonstrates such examples.

#### 3.2.2. Effect of Rhythm Type and Heart Rate on Visualization Quality

*Effect of rhythm type.*

The distribution of visualization categories by rhythm type is presented in Table 10.

Table 10 shows the proportion of all 74 patients for each visualization category across seven cardiac veins: CS trunk, middle vein, posterior vein, posterolateral vein, lateral vein, anterolateral vein and anterior vein. Data are presented separately for three rhythm groups: sinus rhythm, atrial fibrillation and right ventricular pacing. Based on Table 9, it was determined that there were no patients with a score of 0 (the tributary is not visualized). Therefore, Table 10 does not include score of 0.

Logistic regression analysis demonstrated no statistically significant effect of rhythm type on the probability of vein absence for any vein (all *p* > 0.01). The CS trunk was fully visualized (score 2 points in Table 9) in all 74 patients (100%); therefore, statistical analysis was not performed for it. For the remaining veins, the Kruskal–Wallis test was performed (Table A1). Statistically significant differences between rhythm groups were observed only for the posterolateral vein (χ^2^ = 7.00, *p* = 0.030). However, after Bonferroni correction for multiple comparisons, the difference did not reach statistical significance (*p* = 0.181).

*Effect of heart rate.*

Spearman’s rank correlation analysis revealed no statistically significant correlations between heart rate and visualization quality for any vein (Table A2).

#### 3.2.3. Multivariable Analysis

**Figure 5:**
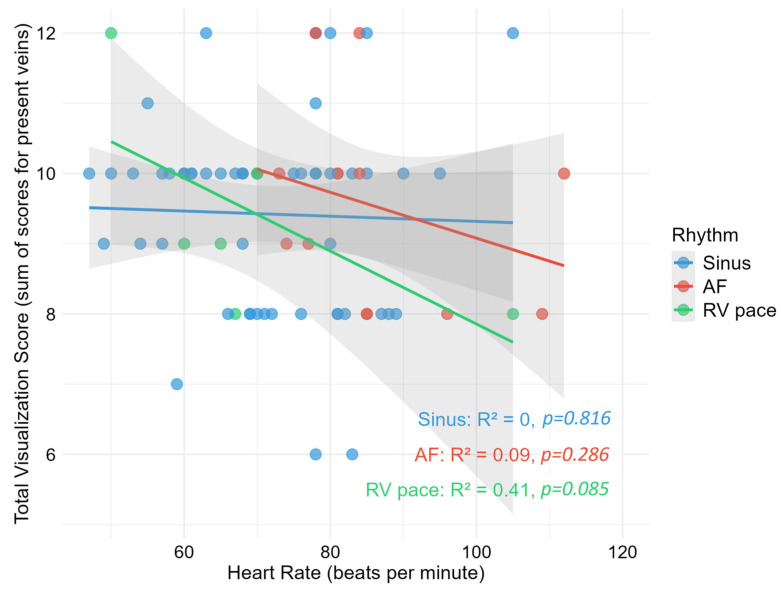
Scatter plot showing the association between heart rate and total venous visualization score. Colored lines represent linear regression fits with 95% confidence intervals for each rhythm group: sinus (sinus rhythm), AF (atrial fibrillation), and RV pace (right ventricular pacing). R^2^ (coefficient of determination) indicates the proportion of variance in the total score explained by heart rate; the *p*-value indicates the statistical significance of the linear relationship. No significant linear association was observed in any rhythm group.

Ordinal logistic regression, accounting for the simultaneous effects of rhythm type and heart rate, revealed no statistically significant independent predictors of visualization quality for any of the analyzed veins (*p* > 0.01 for all predictors) (Figure 5).

The total score on the Y-axis in Figure 5 represents the sum of visualization scores across all seven veins, calculated only for anatomically present veins. Visualization is graded as follows: 0 = not visualized; 1 = partially visualized; and 2 = fully visualized. Anatomically absent veins, assigned a score of −1, were excluded from the calculation. Each point represents an individual patient. For example, if the CS trunk and the middle vein are fully visualized in a patient, each receiving a score of 2, while the anterolateral and the anterior vein are partially visualized, each receiving a score of 1, and no other anatomical tributaries are present, the total score on the Y-axis in Figure 5 for this particular patient will be 6 (2 + 2 + 1 + 1).

### 3.3. Assessment of Parameter Dependencies

#### 3.3.1. Evaluation of Radiation Dose Dependency on Body Mass Index

Total DLP positively correlated with body mass index (BMI) (r_s_ = 0.7, *p* < 0.001, r_s_^2^ = 0.488). BMI accounted for 48.8% of the rank variability in total DLP.

DLP without LungLowDose thorax series also positively correlated with BMI (r_s_ = 0.68, *p* < 0.001, r^2^ = 0.458), indicating that 45.8% of the variation in the ranks was explained by BMI.

Total CTDIvol demonstrated a moderate positive relationship with BMI (r_s_ = 0.69; *p* < 0.001). The coefficient of determination (r_s_^2^ = 0.476) indicated that BMI accounted for approximately 47.6% of the variance in the ranks of CTDIvol.

CTDIvol (measured without LungLowDose thorax series) also demonstrated a significant moderate positive correlation with BMI (r_s_ = 0.69, *p* < 0.001, r_s_^2^ = 0.477), indicating that BMI accounted for 47.7% of the variance in the ranks of CTDIvol.

In general, DLP and CTDIvol values demonstrated moderately positive correlation where higher BMI was associated with increased radiation dose.

#### 3.3.2. Determination of the Dependence of Time-to-Peak in the Ascending Aorta on Other Parameters

**Figure 6:**
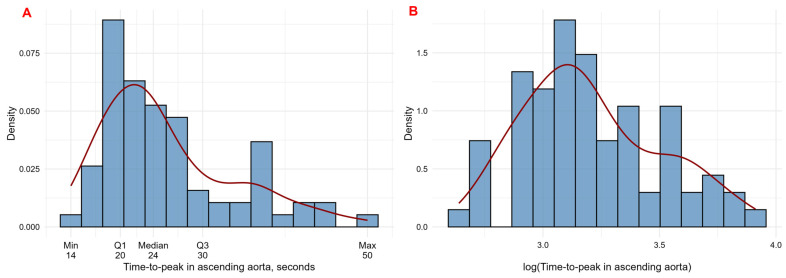
Distribution of time-to-peak contrast opacification in the ascending aorta. (**A**)—the initial data analysis, performed via a histogram with an overlaid density curve, revealed a positive skewness in the distribution. (**B**)—log (time-to-peak in ascending aorta). The distribution was close to normal.

To determine factors associated with aortic time-to-peak contrast opacification, the distribution of the dependent variable was first examined to guide the choice of the regression model. The distribution of time-to-peak in the ascending aorta is presented in Figure 6A, utilizing a histogram with an overlaid density curve to visualize the data. The Shapiro–Wilk test confirmed a statistically significant deviation from a normal distribution (W = 0.904, *p* < 0.0001). Time-to-peak contrast opacification in the aorta after logarithmic transformation is presented in Figure 6B.

The log-normal distribution presented the best fit (AIC = 505.94), outperforming the other alternatives (Table A3).

*GLM fit quality.*

The constructed log-normal GLM demonstrated high goodness-of-fit metrics. The model explained 60.1% of the variance in the log-transformed dependent variable (R^2^ = 0.601, adjusted R^2^ = 0.515). The F-test confirmed the overall statistical significance of the model (F (13.60) =6.96, *p* < 0.001). The correlation between predicted and observed values in the original scale was r_s_ = 0.764, *p* < 0.001, indicating a strong association. The predictive accuracy of the model for time-to-peak in the ascending aorta was assessed on the original scale. The mean absolute error (MAE) was 3.81 s, and the root mean square error (RMSE) was 5.13 s. The relative error, expressed as the coefficient of variation of the RMSE (CV-RMSE), was 19.9%. Furthermore, 71.6% of the predictions were within ±20% of the observed values. Residual diagnostics confirmed model adequacy: the Breusch–Pagan test showed no heteroscedasticity (*p* = 0.497), the Durbin–Watson test showed no residual autocorrelation (DW = 2.08, *p* = 0.634). However, the Shapiro–Wilk test for residuals indicated deviation from normality (*p* = 0.023).

*Regression analysis results.*

Using a statistical significance level of α = 0.01, only 2 out of 10 investigated predictors (20%) demonstrated a statistically significant association with time-to-peak in ascending aorta. In Table A4, the results of the log-normal regression analysis of factors associated with time-to-peak contrast opacification is shown.

HU value and age were identified as significant predictors on the time-to-peak contrast opacification in the ascending aorta based on the Table A4. A one HU increase was associated with an average 0.4% decrease in time-to-peak (95% CI: −0.6% to −0.2%). An increase in patient age by 1 year was associated with an average 0.8% increase in time-to-peak in the ascending aorta (95% CI: 0.3% to 1.3%).

**Figure 7:**
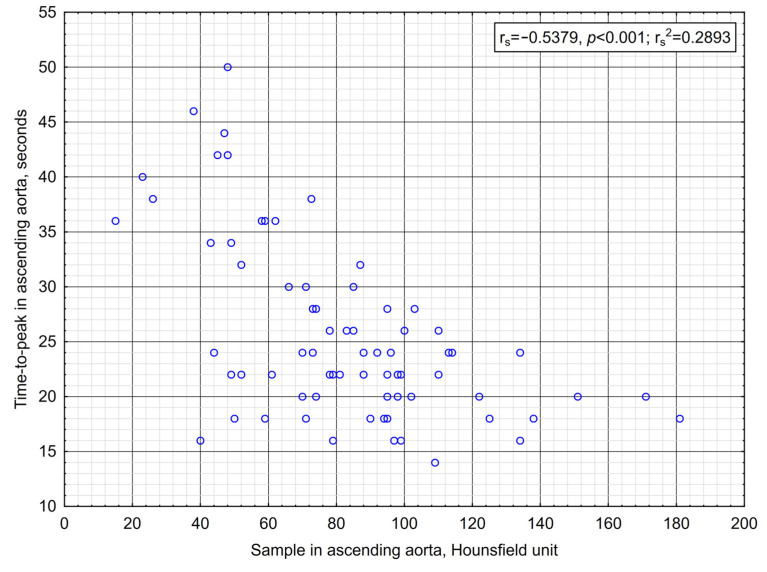
Scatter plot presenting the Spearman correlation between Hounsfield unit and the time-to-peak contrast opacification in ascending aorta.

Next, Figure 7 presents a scatter plot illustrating the relationship between the HU sample and the time-to-peak contrast opacification in the ascending aorta based on the Spearman rank correlation coefficient.

**Figure 8:**
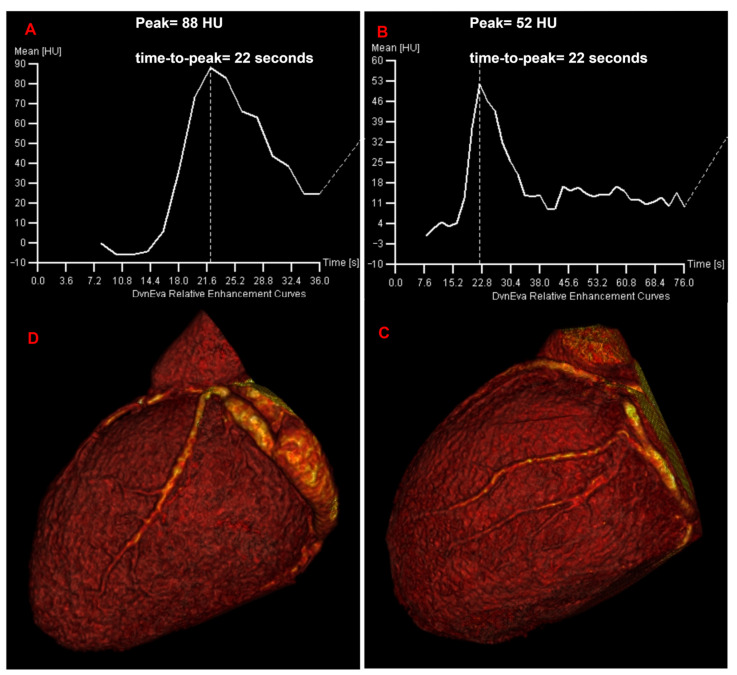
Variation in Hounsfield unit (HU) at identical time-to-peak contrast opacification in ascending aorta. (**A**)—case N1, HU= 88 at the time-to-peak= 22 s; (**B**)—case N2, HU= 52 at the time-to-peak= 22 s; (**C**)—case N2 with final view of coronary sinus veins; (**D**)—case N1 with final view of coronary sinus veins.

A moderate negative correlation was observed between HU and aortic time-to-peak contrast opacification (Spearman’s r_s_ = −0.54, *p* < 0.001; Figure 7). This finding indicated that higher HU values tend to be associated with shorter aortic time-to-peak, although the relationship is not strong. The squared Spearman’s rank correlation coefficient (r_s_^2^ = 0.289) suggested that only 28.9% of the variance in ranks was accounted for by the association between HU value and time-to-peak contrast opacification in the ascending aorta. For an example, Figure 8 illustrated that at equal values of the time-to-peak enhancement in the ascending aorta; the HU values vary.

Also, a weak positive correlation was observed between age and aortic time-to-peak contrast opacification (Spearman’s r_s_ = 0.29, *p* < 0.001). This finding indicated that increasing age tends to be associated with a longer time-to-peak, although the relationship was weak. The coefficient of determination (r_s_^2^ = 0.084) suggested that only 8.4% of the variance in the ranks was shared between the two variables, reflecting a weak association despite its statistical significance.

## 4. Discussion

The primary finding of this study is that the proposed and rigorously detailed MSCT protocol for CS veins imaging provides optimal visualization of at least main trunk and first-order tributaries in a heterogeneous cohort of 74 adult patients meeting Class I and IIa indications for CRT. CS veins anatomy was variable. Using this protocol, we received valuable information that CS venous tree was unsuitable for endovascular LV lead placement in 8 (10.8%) of 74 patients, who subsequently underwent alternative pacing strategies. Chen et al. also reported that in up to 10% of cases, cannulation of the CS is impossible due to abnormalities [10]. Importantly, no significant difference was revealed in visualization quality for all veins in rhythm groups. Heart rate also showed no significant correlation with visualization quality for any vein. Finally, multivariable proportional odds logistic regression confirmed that neither rhythm type nor heart rate emerged as independent predictors of visualization quality for any vein, consistent with the univariable findings. Therefore, our proposed protocol remains independent of heart rhythm and heart rate.

The current study utilizes a test bolus approach previously described [19]. Our distinguishing feature is that we always perform the scanning at the level of the carina, which corresponded to the ascending aorta. We never utilize scanning at the level of the CS, as this carries the risk of obtaining an automated test bolus graph with multiple peaks, noise, and difficulties in interpretation. As demonstrated in Figure 7, the r_s_^2^ coefficient indicated that only 28.9% of the variance in ranks was explained by the association between HU value and time-to-peak contrast opacification in ascending aorta. This implies that patients with similar aortic time-to-peak contrast opacification may exhibit considerably different HU values. Consequently, a fixed HU threshold for bolus tracking may be unreliable across individuals. As the test bolus technique does not rely on a predetermined HU threshold and instead provides an individualized assessment of contrast timing, it represents a more robust approach. Also, we did not observe any patient-specific clinical parameters that affect the time-to-peak contrast opacification. These findings further underscore that the aortic time-to-peak is a highly individualized parameter that can be reliably determined only through a test bolus.

It is important to emphasize that in our protocol test bolus acquisition is initiated only 8 s after the start of contrast administration. This delay is intended to allow the contrast medium sufficient time to reach the aorta, thereby avoiding unnecessary radiation exposure from premature scans. Furthermore, the number of repetitive scans is consistently set to 23 to ensure that the aortic peak enhancement is captured, even in cases where it might be significantly delayed. Notably, the sequence can be manually terminated if the peak opacification is clearly visualized on the monitor in real time.

The next key point concerns the final scanning delay, which is designed to achieve optimal opacification of the CS veins. This delay is calculated as the time-to-peak enhancement in the ascending aorta (determined during the test bolus phase) plus 20 s. This pause is essential to permit adequate accumulation of contrast medium in the venous system. The literature review confirms that an identical 20 s value was employed in a prior study involving a small patient group [19].

Mlynarski R et al. documented that the optimal time for reconstruction should be performed during diastolic phases 30–50% RR interval with retrospective gating despite the higher dose of radiation [16]. In another study, prospective gating with trigger image acquisition at end-systole was employed in a small patient group [15]. With regard to ECG gating, we follow the widely accepted concept that the prospective approach is associated with a lower radiation dose [38]. Therefore, only prospective gating is used. We set phase manually in the range of 200–400 ms for the MSCT series. Manual phase optimization in our variant is selected to achieve maximum image clarity considering the patient’s individual rhythm characteristics and without high dose of radiation.

In the present study, an additional non-contrast LungLowDose series is performed. This torso information is employed to also determine the late activation zone using ECGI in patients before CRT, following previously established works [13,25,26]. Based on the given data, it is possible to compare the target vein and the zone of late activation. This additional non-contrast image series leads to an increase in DLP values. There is a concept known as Diagnostic Reference Level (DRL) [39,40]. Consequently, the dose received during the MSCT can be compared with the DRL value. Analyzing the literature, we were unable to find a specific DRL for CS veins scanning. However, we found that for coronary computed tomography angiography with prospective gating, the DRL for the primary indicator, DLP, is 210 mGy×cm [41]. In our current study, it is found that the DLP, excluding the torso series, is 145.2 [105.7; 217.8], while the DLP including the torso series is 164.5 [116; 237]. Overall, even when accounting for the torso series, our total DLP dose remains within the DRL limit. Dose exceedance occurs in individuals with an increased BMI calculated from height and weight parameters. All DLP values demonstrate a moderately positive correlation, with higher BMI being associated with increased radiation dose. This finding is consistent with the published literature [42]. As previously noted, the non-contrast torso series also contributed to the increase in the total DLP. In this regard, in routine practice, when evaluating only the CS veins, this series may be omitted following the ALARA principle, “as low as reasonably achievable” [41].

We also performed a literature search to identify studies reporting radiation exposure during CS contrast enhancement. In the study by Malagò et al., which employed retrospective ECG gating, the estimated effective radiation dose had a median value of 6.7 millisieverts, with the upper value reaching 12.3 millisieverts [14]. Using a conversion coefficient of 0.014, these values can be converted into DLP values, corresponding to a median value of 478 mGy×cm, with the upper value reaching 878 mGy×cm. By comparison, in our study, the DLP achieved with prospective ECG gating, even when including the torso series, was approximately three times lower, with a median value of 164.5 [116; 237] mGy×cm. This further underscores that prospective ECG gating should be used for CS contrast-enhanced imaging.

The contrast agent used should also be mentioned. While an agent with high iodine concentration undoubtedly provides superior image quality, the use of a standard concentration should be considered. In our study we use only agent with an iodine concentration of 300 mg I/mL. The resulting image quality demonstrates that this standard iodine concentration is sufficient for adequate CS veins visualization. The total contrast agent dose in our protocol reaches 115 mL, which is consistent with the established range of 100–120 mL reported in previously published studies [11,13].

Last but not least, contrast agent administration is performed via the antecubital vein. The literature suggests an injection rate of 5–6 mL per second [19,20]. However, not every candidate has a vein suitable for a catheter that can accommodate such a high flow rate. In our protocol, we use a rate that does not exceed 4.5 mL per second, which allows the use of a smaller-diameter catheter in all patients.

*Limitations*

First, this was a single-center prospective study. On the other hand, a diverse group of patients with Class I and IIA indications for CRT was included. This provides confidence that the protocol is applicable to all CRT candidates.

Second, we acknowledge that a formal a priori power calculation was not performed. The effect-size estimates derived from this 74 patients’ cohort will inform the sample-size determination of a future adequately powered confirmatory study.

Third, this study was conducted using a single MSCT scanner (SOMATOM Force, Siemens Healthineers, Germany). Although the test-bolus protocol is based on patient-specific contrast kinetics and should therefore be conceptually applicable across vendors, scanner-dependent acquisition and reconstruction parameters may affect reproducibility. Consequently, our findings cannot be directly extrapolated to other manufacturers without dedicated validation.

Fourth, the provided protocol involved the use of a dual-syringe injector to administer a contrast-saline mixture during the final pass. It is assumed that dual-syringe injectors for mixed administration of contrast and saline are predominantly available in modern clinics.

Fifth, a limitation of this study is that all images were estimated by a single operator with 15 years of experience. Consequently, inter-rater variability was not evaluated, and the reproducibility of the scoring system across observers with varying levels of expertise remains to be established. Future multi-reader studies, including formal assessment of inter- and intra-observer agreement (e.g., weighted kappa or intraclass correlation coefficients), are warranted to confirm the robustness and broader applicability of this grading system.

## 5. Conclusions

A personalized, detailed MSCT protocol for visualization of the coronary sinus veins is presented. The key elements of this protocol include the use of a test bolus in the ascending aorta to calculate the final scan delay, prospective ECG gating to accommodate variations in cardiac rhythm and heart rate in individual patients, a contrast medium with a standard iodine concentration of 300 mg I/mL, and an injection rate not exceeding 4.5 mL/s. Venous visualization quality is independent of heart rate and cardiac rhythm during the examination. Multi-vendor, multi-center studies are warranted to confirm the generalizability and external validity of the proposed MSCT protocol.

## Figures and Tables

**Table 1:** Parameters for performing a Topogram.

Parameter	Required Value
Topogram direction	Top (anterior–posterior)
Topogram length	512 mm
Scanning direction	Craniocaudal
AP instruction	Inspiration

**Table 2:** Parameters for performing a non-contrast LungLowDose series.

Parameter	Required Value
Scan tab	
kVp	Care kV (ref. kV 100)
Effective mAs	CareDose
Quality Reference mAs	25
Time (Rotation)	0.5 s
Collimation	184 × 0.6 mm
Scan Direction	Craniocaudal
AP instruction	Inspiration
Recon tab	
Recon region	Wide
Recon job type	Axial
Slice Thickness	1 mm
Increment	1 mm
ADMIRE	On
Strength	4
Kernel	Br36
FAST	On
Window	Mediastinum
Mirroring	None

**Table 3:** Test bolus features.

Parameter	Required Value
Scan tab	
kV	100
mAs	23
Number of scans	23
Delay	8 s
Scan time	Full 0.25 s
Cycle time	2 s
Recon tab	
Slice	10 mm (1 × 10 mm)
Kernel	Br36
Window	Cardiac

**Table 4:** Contrast agent and saline during test bolus.

Phase	Agent	Speed Flow, mL/s	Volume, mL
A	Contrast	4.5	10
B	Saline	4.5	50

**Table 5:** DS-CorAdSeq features.

Parameter	Required Value
Scan tab	
kVp	Care kV (ref. kV 100)
Dose saving optimized for:	Set to number 11 (angio)
Effective mAs	CareDose
Quality Reference mAs	288
Time (Rotation)	0.25 s (flex)
Collimation	152 × 0.6 mm
Scan Direction	Craniocaudal
AP instruction	Inspiration
Trigger tab	
Scan	Manual
Range	200–400 ms
Pulsing	200–400 ms
Unit	ms
BestPhase	Manual
Phase start	250 ms
Unit	ms
Recon	Quick 66 ms
Recon tab	
Slice Thickness	1 mm
Increment	1 mm
ADMIRE	On
Strength	4
Kernel	Br34
FAST	On
Window	Cardiac

**Table 6:** Contrast agent and saline during DS-CorAdSeq series.

Phase	Agent	Speed Flow, mL/s	Volume, mL
A	Contrast	4.5	50
A	Contrast	3.5	30
A + B	Contrast + Saline	3.5	50 *

*—Proportion of contrast/saline is 1:1 (25/25 mL).

**Table 7:** Metrics of the study population.

Parameter	Value
Total number of cases, *n*	74
Male/female, *n* (%)	44 (59.5%)/30 (40.5%)
Age, years	64 (27; 84) *
Height, cm	174 (147; 198) *
Weight, kg	82 (44; 153) *
Body mass index, kg/m^2^	27.3 (17.2; 40.3) *
Genesis, DCM/IHD/CHD, *n* (%)	34 (45.9%)/33 (44.6%)/7 (9.5%)
NYHA II/III/IV functional class, *n* (%)	42 (56.8%)/29 (39.2%)/3 (4%)
Own rhythm sinus/AF/RV pacing, *n* (%)	52 (70.3%)/14 (18.9%)/8 (10.8%)
QRS duration, ms	178 (121; 240) *
QRS pattern LBBB/IVCD/pacing, *n* (%)	56 (75.7%)/9 (12.15%)/9 (12.15%)
LV EF, %	29 (10; 40) *
LV EDV, mL	199 (63; 444) *
LV ESV, mL	144 (42; 328) *
Recommendation class CRT I/IIa, *n* (%)	42 (56.8%)/32 (43.2%)

*—median (min; max); AF—atrial fibrillation; CRT—cardiac resynchronization therapy; DCM—dilated cardiomyopathy; CHD—congenital heart disease; IHD—ischemic heart disease; IVCD—intraventricular conduction disturbance; LV EF—left ventricle ejection fraction; LV EDV—left ventricle end-diastolic volume; LV ESV—left ventricle end-systolic volume; NYHA—New York heart association; RV—right ventricle pacing.

**Table 8:** Calculated tomography metrics.

Parameter	Value				
	Median	Min	Max	Q1	Q3
Total DLP, mGy×cm	164.5	54	754	116	237
DLP without LungLowDose torso series, mGy×cm	145.2	49.5	728	105.7	217.8
Delta DLP, mGy×cm	14.2	0.19	71	7.7	22
Total CTDIvol, mGy	23.5	7.6	57.25	20.9	28.2
CTDIvol without LungLowDose torso series, mGy	23.1	7.4	56.6	20.6	27.8
Delta CTDIvol, mGy	0.3	0.06	4.5	0.16	0.42
Heart frequency, bpm	73.5	47	112	63	82
Time-to-peak contrast opacification in ascending aorta, seconds	24	14	50	20	30
Sample in ascending aorta, HU	80	15	181	59	98
Final delay for contrast venous phase, seconds	44	34	70	40	50

bpm—beats per minute; Computed Tomography Dose Index Volume—CTDIvol; DLP—Dose Length Product; Delta—median difference; HU—Hounsfield unit; mGy—milligray; mGy×cm—milligray-centimeters; Q1—low quartile; Q3—upper quartile.

**Table 9:** Vein scoring.

Score	CS Trunk	Middle Vein	Posterior Vein	Posterolateral Vein	Lateral Vein	Anterolateral Vein	Anterior Vein
2	74 (100%)	73 (98.7%)	42 (56.8%)	31 (41.9%)	34 (45.9%)	17 (23%)	68 (91.9%)
1	0 (0%)	1 (1.3%)	5 (6.7%)	1 (1.3%)	5 (6.8%)	4 (5.4%)	6 (8.1%)
0	0 (0%)	0 (0%)	0 (0%)	0 (0%)	0 (0%)	0 (0%)	0 (0%)
−1	0 (0%)	0 (0%)	27 (36.5%)	42 (56.8%)	35 (47.3%)	53 (71.6%)	0 (0%)

−1 point—the vein is absent in the patient; 0 point—the tributary is not visualized; 1 point—the tributary is partially visualized (not throughout its entire length); 2 points—the tributary is fully visualized (throughout its entire length). CS—coronary sinus. Middle (or posterior interventricular) vein. Anterior (or anterior interventricular vein). Numbers indicate the absolute patient count (n) and the corresponding percentage (%).

**Table 10:** Distribution of venous visualization categories across different cardiac rhythm types.

Score	CS Trunk	Middle Vein	Posterior Vein	Posterolateral Vein	Lateral Vein	Anterolateral Vein	Anterior Vein
Sinus rhythm							
2	52 (100%)	52 (100%)	28 (53.8%)	25 (48.1%)	23 (44.2%)	13 (25%)	46 (88.5%)
1	0 (0%)	0 (0%)	1 (2%)	0 (0%)	2 (3.8%)	3 (5.8%)	6 (11.5%)
−1	0 (0%)	0 (0%)	23 (44.2%)	27 (51.9%)	27 (52%)	36 (69.2%)	0 (0%)
Atrial fibrillation							
2	14 (100%)	13 (92.9%)	9 (64.3%)	3 (21.4%)	7 (50%)	3 (21.4%)	14 (100%)
1	0 (0%)	1 (7.1%)	3 (21.4%)	1 (7.1%)	2 (14.3%)	1 (7.2%)	0 (0%)
−1	0 (0%)	0 (0%)	2 (14.3%)	10 (71.5%)	5 (35.7%)	10 (71.4%)	0 (0%)
Right ventricular pacing							
2	8 (100%)	8 (100%)	5 (62.5%)	3 (37.5%)	4 (50%)	1 (12.5%)	8 (100%)
1	0 (0%)	0 (0%)	1 (12.5%)	0 (0%)	1 (12.5%)	0 (0%)	0 (0%)
−1	0 (0%)	0 (0%)	2 (25%)	5 (62.5%)	3 (37.5%)	7 (87.5%)	0 (0%)

−1 point—the vein is absent in the patient; 1 point—the tributary is partially visualized (not throughout its entire length); 2 points—the tributary is fully visualized (throughout its entire length). CS—coronary sinus. Middle (or posterior interventricular) vein. Anterior (or anterior interventricular vein). Numbers indicate the absolute patient count (n) and the corresponding percentage (%) inside category.

## Data Availability

Raw dicom data from MSCT is available on a reasonable request.

## Abbreviations

The following abbreviations are used in this manuscript:

AF	Atrial fibrillation
BMI	Body mass index
CRT	Cardiac resynchronization therapy
CS	Coronary sinus
CTDIvol	Computed Tomography Dose Index Volume
DCM	Dilated cardiomyopathy
DLP	Dose Length Product
DRL	Diagnostic Reference Level
ECG	Electrocardiography
ECGI	Electrocardiographic imaging
HU	Hounsfield unit
IHD	Ischemic heart disease
mg I/mL	Milligrams of iodine per milliliter
milligray	mGy
milligray-centimeters	mGy×cm
mL	Milliliters
mL/s	Milliliters per second
MSCT	Multislice computer tomography
ms	Milliseconds
mm	Millimeters
LV	Left ventricle
LV EDV	Left ventricle end-diastolic volume
LV ESV	Left ventricle end-systolic volume
LV EF	Left ventricle ejection fraction
r_s_	Spearman’s correlation coefficient

## Appendix A

**Table A1:** Kruskal–Wallis test results for visualization quality.

Vein	χ^2^	*p*	*p* (Adjusted)
Anterior	2.73	0.256	1.000
Anterolateral	0.31	0.855	1.000
CS trunk *	-	-	-
Lateral	1.42	0.491	1.000
Middle	4.29	0.117	0.702
Posterior	4.32	0.116	0.696
Posterolateral	7.00	0.030	0.181

* Coronary sinus trunk was fully visualized in all patients; statistical analysis was not performed.

**Table A2:** Correlation between visualization quality and heart rate.

Vein	n	r_s_	*p*
Anterior	74	0.095	0.420
Anterolateral	21	−0.281	0.217
CS trunk *	74	-	-
Lateral	39	−0.034	0.837
Middle	74	−0.178	0.129
Posterior	47	−0.046	0.760
Posterolateral	32	−0.224	0.218

* Coronary sinus trunk was fully visualized in all patients; correlation was not calculated.

**Table A3:** Comparison of distribution goodness-of-fit statistics.

Distribution	AIC	BIC
Log-normal	505.94	510.55
Gamma	509.93	514.54
Normal	522.67	527.28
Weibull	523.53	528.13

AIC—Akaike Information Criterion; BIC—Bayesian Information Criterion.

**Table A4:** Results of log-normal regression analysis.

Predictor	β (Log Scale)	Standard Error	t-Value	*p*-Value
Intercept	2.6917	0.1964	13.71	<0.001
Age	0.0078	0.0023	3.34	0.0014
HU in aorta	−0.0040	0.0010	−4.09	<0.001
Body mass index	0.0078	0.0053	1.47	0.147
QRS complex duration	0.0007	0.0010	0.74	0.464
Heart frequency	0.0018	0.0021	0.85	0.397
LV EF	−0.0068	0.0055	−1.24	0.220
Gender (female vs. male)	0.0096	0.0532	0.18	0.858
Own rhythm (AF vs. sinus)	0.0331	0.0813	0.41	0.685
Own rhythm (pace vs. sinus)	0.0214	0.0905	0.24	0.814
Genesis (CHD vs. DCM)	−0.0030	0.0985	−0.03	0.976
Genesis (IHD vs. DCM)	0.0092	0.0548	0.17	0.868
NYHA (class III vs. II)	0.1156	0.0595	1.94	0.056
NYHA (class IV vs. II)	0.1935	0.1644	1.18	0.245
